# The Neglected Price of Pediatric Acute Kidney Injury: Non-renal Implications

**DOI:** 10.3389/fped.2022.893993

**Published:** 2022-06-30

**Authors:** Chetna K. Pande, Mallory B. Smith, Danielle E. Soranno, Katja M. Gist, Dana Y. Fuhrman, Kristin Dolan, Andrea L. Conroy, Ayse Akcan-Arikan

**Affiliations:** ^1^Division of Critical Care Medicine, Department of Pediatrics, Baylor College of Medicine, Texas Children's Hospital, Houston, TX, United States; ^2^Division of Pediatric Critical Care Medicine, Department of Pediatrics, University of Washington, Seattle, WA, United States; ^3^Harborview Injury Prevention and Research Center, University of Washington, Seattle, WA, United States; ^4^Section of Nephrology, Departments of Pediatrics, Bioengineering and Medicine, University of Colorado, Aurora, CO, United States; ^5^Division of Cardiology, Department of Pediatrics, Cioncinnati Children's Hospital Medical Center, University of Cincinnati School of Medicine, Cincinnati, OH, United States; ^6^Division of Critical Care Medicine, Department of Pediatrics, UPMC Children's Hospital of Pittsburgh, Pittsburgh, PA, United States; ^7^Division of Nephrology, Department of Pediatrics, UPMC Children's Hospital of Pittsburgh, Pittsburgh, PA, United States; ^8^Division of Critical Care Medicine, Department of Pediatrics, University of Missouri Kansas City, Children's Mercy Hospital, Kansas City, MO, United States; ^9^Ryan White Center for Pediatric Infectious Disease and Global Health, Department of Pediatrics, Indiana University School of Medicine, Indianapolis, IN, United States; ^10^Division of Nephrology, Department of Pediatrics, Baylor College of Medicine, Texas Children's Hospital, Houston, TX, United States

**Keywords:** acute kidney injury, pediatric critical care medicine, acute lung injury, cardiac dysfunction, functional status, growth, neurologic injury, immunoparalysis

## Abstract

Preclinical models and emerging translational data suggest that acute kidney injury (AKI) has far reaching effects on all other major organ systems in the body. Common in critically ill children and adults, AKI is independently associated with worse short and long term morbidity, as well as mortality, in these vulnerable populations. Evidence exists in adult populations regarding the impact AKI has on life course. Recently, non-renal organ effects of AKI have been highlighted in pediatric AKI survivors. Given the unique pediatric considerations related to somatic growth and neurodevelopmental consequences, pediatric AKI has the potential to fundamentally alter life course outcomes. In this article, we highlight the challenging and complex interplay between AKI and the brain, heart, lungs, immune system, growth, functional status, and longitudinal outcomes. Specifically, we discuss the biologic basis for how AKI may contribute to neurologic injury and neurodevelopment, cardiac dysfunction, acute lung injury, immunoparalysis and increased risk of infections, diminished somatic growth, worsened functional status and health related quality of life, and finally the impact on young adult health and life course outcomes.

## Introduction

Acute kidney injury (AKI) is common in critically ill children, occurring in up to 25% of the general pediatric intensive care unit (PICU) population and up to 40–60% of the pediatric cardiac intensive care unit (CICU) population (1–6). Although most studies are in children from high-income countries in the context of intensive care settings, AKI is a global problem associated with considerable morbidity and mortality (7). Once thought to be an isolated syndrome, emerging evidence suggests that AKI has far reaching effects in the body that affect short and long term outcomes in critically ill children. Recent evidence suggests that, with AKI, molecular and biologic mediators are involved in organ crosstalk at a cellular and genomic level in critical illness.

Hospitalized children who develop AKI have increased morbidity and mortality. Specifically, patients who develop AKI have longer length of mechanical ventilation, longer ICU and hospital lengths of stay, as well as increased health resource utilization (4, 8–13). AKI is an independent risk factor for mortality in pediatric patients with critical illness, after cardiac surgery, sepsis, acute respiratory distress syndrome, recent surgery, and oncologic disorders (6, 9, 14–17). Furthermore, the risk of mortality extends beyond the hospital admission: patients with AKI during acute illness have higher mortality rates years after discharge compared to patients without AKI (18, 19). The independent association of AKI with morbidity and mortality, with or without the presence fluid overload, may be mediated by the effects of AKI on other organ systems.

Current standardized definitions of AKI only focus on rapid creatinine elevations from baseline and varying degrees of oliguria with an “or” logic. By the Kidney Disease: Improving Global Outcomes (KDIGO) criteria, AKI severity is stratified based on fold-increase of creatinine or duration of oliguria (20). These definitions are agnostic to etiology, course, rate and degree of recovery, and timing of onset in relation to critical illness. Evidence is rapidly accumulating to indicate that severity is not the only dimension that has outcome implications; rather, timing of onset, duration, number of episodes, and rate of recovery, have discrete impacts on organ and global outcomes (21–25).

**Figure 1:**
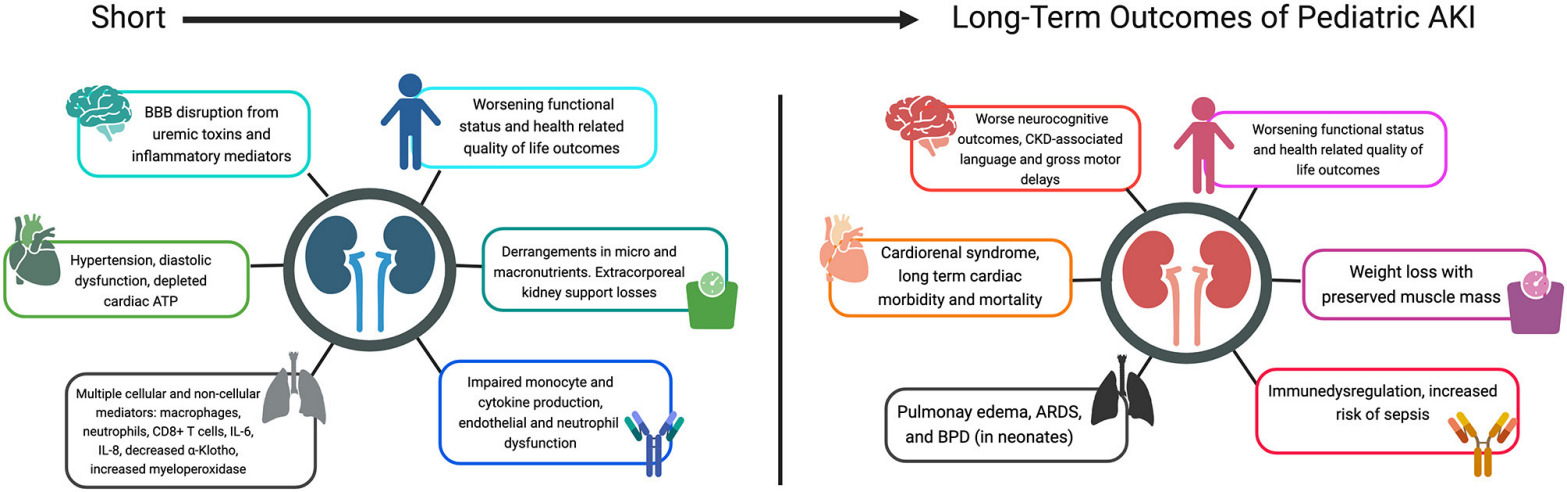
Short and long term outcomes of pediatric AKI. BBB, blood-brain-barrier; ATP, adenosine triphosphate; CD8+, cluster of differentiation 8; IL-, interleukin; CKD, chronic kidney disease; ARDS, acute respiratory distress syndrome; BPD, bronchopulmonary dysplasia.

Renal recovery after AKI episodes is not always complete. Certain risk factors, such as extent of pre-morbid kidney health, repeat events, and underlying risk factors contribute to impaired recovery with long term consequences (22, 23, 25). About 10% of AKI survivors develop chronic kidney disease (CKD) (26–29). Emerging preclinical evidence suggests that organ crosstalk in AKI leads to short and long term adverse events on all organ systems, with remote consequences (Figure 1) (30). For the practicing intensivist, an understanding of the non-renal effects of AKI can help them tailor treatment strategies and follow-up plans that are focused on preventing these sequelae and ultimately, death. This review aims to discuss the non-renal effects of AKI both in the acute critical illness and long-term recovery phases.

## AKI and the Effects on Other Organ Systems

### AKI and the Brain

In recent years, an intriguing crosstalk between the kidneys and the brain has been discovered. In a murine model of ischemia/reperfusion (I/R) AKI, the blood-brain-barrier was disrupted with increased levels of proinflammatory chemokines in the cerebral cortex and corpus callosum, as well hippocampal neuronal dysfunction and apoptosis 24 h later (31, 32). These changes translated into reduced cognitive performance and memory loss in the mice (32, 33). There is accumulating evidence from diverse pediatric cohorts supporting the concept of kidney-brain crosstalk leading to long-term neurocognitive dysfunction that extends beyond critically ill populations and includes children hospitalized with community-acquired AKI (29, 32, 34, 35).

Mechanistically, endothelial activation in the context of AKI may contribute to blood-brain-barrier dysfunction resulting in brain injury through exposure to uremic toxins and inflammatory mediators. These changes, in turn, may contribute to neurodevelopmental delay in critically ill children. One pathway implicated in AKI-related brain injury is the angiopoietin (angpt)-Tie-2 axis that regulates endothelial integrity. Angpt-1 is a growth factor that maintains a quiescent resting state of the vascular endothelium, whereas its counterpart, Angpt-2 is rapidly released by activated endothelium (36). Angpt-2 primes the endothelium to respond to inflammatory cytokines, upregulates cellular adhesion molecules, and promotes remodeling, but can also lead to vascular leak and endothelial cell apoptosis (37). A higher ratio of Angpt-2/Angpt-1 is thought to contribute to the pathogenesis of organ injury seen in AKI, critical illness, cardiopulmonary bypass, and severe malaria (38–40). Elevated Angpt-2 levels and the resultant altered Angpt-2/Angpt-1 ratio is linked to increased vascular permeability and inflammation that compromise the tight junctions of the blood-brain-barrier (38). This imbalance is independently associated with worse cognitive function in patients with severe malaria who develop AKI with deficits in fine and gross motor skills, visual reception, receptive and expressive language, and learning (38). Other insults such as inflammatory processes, neurotransmitter derangement, and oxidative injury that are associated with cognitive impairment have also been seen in critically ill children with AKI (41).

Emerging evidence confirms a link between AKI and worse neurocognitive outcomes. In a large retrospective multicenter PICU database study of almost 30,000 patients from 24 PICUs, it was found that cognitive disability was present in 12.2% of patients who received continuous renal replacement therapy (CRRT). This was consistent with a decline in cognitive function at hospital discharge (OR 1.76) (42). Recurrent AKI after distinct cardiac surgeries in children with congenital heart disease has been shown to be associated with worse neurodevelopmental outcomes, specifically in language, motor, and cognitive domains, and most pronounced in the language domain (34). In a cohort of pediatric patients with diabetic ketoacidosis, after adjusting for demographics and severity, those who developed AKI had lower IQ scores and worse short term memory 6 months after recovery (35).

AKI is common in patients with malaria and is associated with neurologic deficits in several patient populations (19, 25, 29, 34). In prospective cohort studies of children with malaria, 25–37% of children with AKI had acute neurologic deficits compared to 2–13% of those patients without AKI. Risk factors for neurologic deficits in children with severe malaria associated AKI include elevated blood urea nitrogen and persistent AKI (25). In persons with malaria, AKI, and acute neurologic deficits, neurocognitive differences persisted for up to 2 years following illness. The relationship was independent of socioeconomic and nutritional status, parental and child education, enrichment in the home environment, and disease severity during hospitalization (29). Furthermore, in children ≥6 years of age with malaria, those who developed AKI while hospitalized had worse scores in socio-emotional function and behavioral regulation up to 2 years following illness (43).

Finally, AKI is a risk factor for CKD which has been associated with poor neurocognitive outcomes including language and gross motor skills, as well as executive function and decision making abilities (28, 44–46). Lower academic achievement is also present in 34% of children with CKD, with the worst scores in mathematics (47).

### AKI and the Heart

The organ cross-talk between the kidneys and heart has been well-established, with five distinct cardiorenal syndromes currently described (48). Cardiorenal syndrome type 3 (CRS3) specifically encompasses AKI that results in acute cardiac dysfunction. However, the description of CRS3 has not included an evaluation on long-term cardiovascular outcomes after AKI recovery. Growing epidemiological evidence in adults suggest associations of AKI events with long-term cardiovascular morbidity and mortality, even in those who have complete kidney recovery. In adults, after adjusting for confounders, AKI is associated with cardiovascular events, especially heart failure by 1 year after hospital discharge (49–52). The effect of AKI on cardiovascular function in children has not been reported. There are several ongoing retrospective evaluations in discrete populations.

A causal mechanism is yet to be established between AKI and cardiovascular dysfunction in adults (53). Recent reports using a murine model of bilateral ischemia-reperfusion AKI in intact adult C57BLK/6J male mice demonstrated diastolic dysfunction that preceded hypertension and was characterized by abnormal cardiac metabolism and depleted cardiac adenosine 5'-triphosphate (ATP) reserves (53, 54). In fact, the cardiac metabolites affected in AKI were noted to be remarkably similar to that of direct myocardial ischemia (53). Importantly, the cardiovascular dysfunction demonstrated after I/R AKI in mice persisted 1 year after the AKI (54). In the long-term murine AKI model, treatment with ITF2357, a non-specific histone-deacetylase inhibitor, prevented the development of diastolic dysfunction, hypertension and reduced cardiac ATP levels (54). In a secondary analysis of AKI and cardiac outcomes by sex, female mice maintained normal diastolic function and cardiac ATP levels compared to male mice with matched AKI severity. However, female mice developed hypertension and renal fibrosis comparable to male mice (55). Analysis of the cardiac metabolome 1 year after injury implicates sex differences in oxidative stress as a potential mechanism to explain the differential cardiorenal outcomes between males and females. Translational studies are needed to establish the mechanistic derangements of cardiorenal syndrome in humans and to evaluate if there is a protective effect in women. In pediatric patients, the potential role of pubertal status on long-term cardiorenal outcomes also warrants investigation.

### AKI and the Lungs

Cellular and molecular mediators of lung injury have been described in the setting of AKI. The initial response to ischemic and nephrotoxic renal injury is mediated by macrophages and neutrophils and may lead to an unchecked pro-inflammatory state resulting in distant pulmonary injury (56–64). Ischemic renal injury in mice leads to an increase in circulating CD8+ T-cells in the lung along with increased markers of T-cell activation. These mice also had increased levels of caspase-3 which mediates pulmonary epithelial cell apoptosis (65). The non-cellular pro-inflammatory mediators of lung injury include interleukin-6 (IL-6) and interleukin-8 (IL-8). Both molecules are found to be elevated in the serum of patients with AKI and are also implicated in lung injury (66–69).

AKI leads to other changes in the concentrations of circulating mediators that may lead to lung injury as well. Uremic toxins are increased in the setting of AKI and are known to cause endothelial cell dysfunction, increased gene expression of IL-6, and decreased pulmonary sodium clearance via downregulation of aquaporins and sodium-channels resulting in pulmonary edema (66, 70–78). The production of α-Klotho occurs exclusively in the kidney. The absence of this molecule has been associated with emphysematous changes in the lung. Patients with AKI have markedly reduced levels of circulating α-Klotho suggesting it may play a role in protecting the lung from kidney mediated injury (79–90).

Newer animal models have also shown increased markers of inflammatory reactive oxidant species generating myeloperoxidase activity in subjects with AKI compared to sham cohorts up to 14 days after initial injury (30). In a secondary analysis of the Assessment of Worldwide Acute Kidney injury Epidemiology (AWAKEN) retrospective cohort trial, neonates born between 29 and 32 weeks gestation who developed AKI in the Neonatal ICU had a four-fold higher odds of developing moderate or severe bronchopulmonary dysplasia in multivariable analyses (91). Given that premature neonates in this age growth are at a critical point of pulmonary angiogenesis coupled with altered vascular growth factors in AKI, it is hypothesized that the disrupted physiologic processes in AKI may potentiate lung injury in this fragile cohort of patients (91). Furthermore, in infants born at ≥32 weeks gestation, AKI is independently associated with worse lung outcomes including higher likelihood of chronic lung disease and longer dependence on oxygen and respiratory support (92). For an in-detail review of the complex interaction between the lungs and kidney, the readers are referred to Alge et al. (56).

### AKI and the Immune System

More recently, the kidney-immune system cross talk has begun to take shape such that the development of AKI is considered to be an immunocompromised state (93). The concept that the kidney plays a role in immune regulation is not new. In fact, it has now been nearly 2 decades since it was noted that there is impaired monocyte cytokine production in critically ill patients with AKI (94). A secondary analysis of the Program to Improve Care in Acute Renal Disease (PICARD) (95) and a single center study in the United Kindom both demonstrated that patients with AKI experience high rates of infectious complications, including sepsis, occurring at a median of 5 days after AKI diagnosis (96). More recently, these results have been recapitulated in homogenous and heterogenous groups of patients across the age spectrum (97–100). Interestingly, the immune effect of AKI appears to be prolonged, even in the presence of complete recovery of AKI. This was demonstrated in a propensity matched analysis where there was a 4.5-fold greater odds of infection within 30 days of discharge in critically ill adults with complete recovery of AKI (100). The association between AKI and infection remained significant at 31–90 and 91–365 days. In children, the association between AKI and subsequent infection was assessed after the Norwood operation, the most complex palliative procedure for newborns with a single ventricle and ductal dependent systemic blood flow. In this study, after adjusting for confounding variables, the was a 3.6-fold greater odds of subsequent infection in neonates with postoperative AKI (98). In a single center retrospective cohort study, a higher odds of infection in a single center study of 5,000 critically ill children: there was a non-linear increase in risk for sepsis based on AKI severity, with stage 3 AKI patients incurring the greatest risk for sepsis (99). A small single center study of pediatric patients receiving CRRT also found an association with infection, that occurred a median of 11 days after CRRT initiation (101).

Little is known about the mechanisms by which the inflammatory cascade that results from AKI may contribute to the development of subsequent sepsis, and this is certainly the focus of substantial research. In an observational study to examine the impact of renal disease on patients with critical illness, patients with AKI developed a reduction in 7 primary amino acids that have been implicated in endothelial and immune dysfunction (102). New data suggest an interaction between the kidney and intestinal microbiome. The intenstinal microbiota are directy involved in immune homeostasis through regulation and induction of both arms of the immune system (103). In addition, in experimental models, neutrophil function is impaired early on in the evolution of AKI, and uremic toxins, such as resistin, may contribute to immune dysfunction (104). More work is needed to enhance our understanding of the the role AKI plays in the sepsis causal pathway. Until we identify the mechanistic derangements, therapeutic targets are limited. Indeed, for now, we can only anticipate the inevitable septic episodes and provide supportive care.

### AKI and Growth

The impact of AKI on long term growth outcomes has been scarcely described in the pediatric literature. Even after initial recovery from induced I/R AKI in animal models, growth parameters in mice with AKI were affected long-term compared to those without AKI: mice in the AKI cohorts weighed significantly less than healthy and sham controls, which occurred irrespective of sex. There was no apparent reduction in muscle mass, indicating a potential decrease in fat and/or bone (30, 55). Macro- and micronutrient derangements, as well as alterations to vital minerals, vitamins, and growth factors have been described in AKI (105). In fact, AKI is a risk factor for protein-energy debt in critically ill children and might be augmented by extracorporeal kidney support related losses (106–108). A recent review discusses the deleterious impacts of AKI on dysregulation of mineral metabolism and its direct effects on bone health (109). The investigation of anthroprometic outcomes following pediatric AKI is warranted.

### AKI and Sex as a Biological Variable

The NIH released the notice “Consideration of Sex as a Biological Variable in NIH-funded Research” in 2015; however un-pooled gender-based investigations of sex as a biological variable in the study of kidney disease remain lacking. Animal models demonstrate a protective effect of female sex in ischemia-reperfusion AKI (110, 111), however conflicting data remain in humans. Clinically, females do better than males with regards to AKI development, CKD progression, and the need for dialysis treatment in hospital-acquired AKI (112–116). Yet controversies remain—the KDIGO supplemental guidelines state that female sex confers a higher risk in developing AKI after cardiac surgery and nephrotoxin exposure despite several studies by Neugarten et al. demonstrating improved outcomes in women compared to men (116–118). Women tend to have slower progression of CKD compared to men, however some studies present conflicting data, likely owing to the inclusion of a mix of pre- and post-menopausal women (119–121). The effect of pubertal development in boys and girls and its impact on the development of AKI, recovery from AKI, and progression to CKD have yet to be determined.

### AKI and Functional Status, Health-Related Quality of Life

Mortality is not the only important metric to assess the impact of critical illness in childhood. Most children who are admitted to ICU survive their critical illness, albeit with varying degrees of acquired morbidity (122). Functional outcomes of survivors after critical illness are core outcome indicators for clinical care benchmarking, developmental research, and ensuring adequate follow-up post ICU stay (123, 124). Children who have new physical disabilities and limitations may not be able to interact with their environment or participate in school at the level they did prior to their illness. This can result in a decline in their health-related quality of life as well as emotional and social functioning. Long-term outcome cohort studies in the general PICU population such as the Wee-Cover (125) and the Survivor Outcomes Study (126) did not assess the risk of functional declines due to AKI or kidney support. Current evidence linking AKI events and functional outcomes are largely restricted to septic AKI cohorts and CRRT survivors. In a secondary analysis of the cross-sectional epidemiology of sepsis (SPROUT) study, 24% of patients with severe AKI (KDIGO 2 and 3) developed new morbidity compared to only 15% of patients without severe AKI, based on the Pediatric Overall Performance Category (POPC) scale (127). More recently, the Life After Pediatric Sepsis Evaluation (LAPSE) study showed similar results using the more granular Functional Status Scale (FSS). In this study, patients with severe septic AKI were more likely to have new morbidity at hospital discharge compared to patients without kidney injury or stage 1 AKI (8). At the 3-month follow-up, 31% of patients with severe AKI had a decline in health-related quality of life by 25% or more from baseline and was mostly due to declines in physical function (8). The association of renal dysfunction and poor functional outcomes was also seen in a cohort of PICU patients with respiratory failure. The Randomized Evaluation of Sedation Titration for Respiratory Failure (RESTORE) study showed that more patients with global functional decline at 6-month follow-up had renal dysfunction during admission than those without decline at 6-month follow-up (11 vs. 5%) (128).

Children who require CRRT during PICU admission may be at higher risk for developing functional decline after critical illness than patients with AKI that do not require dialysis. In a retrospective review of patients who received CRRT at a single tertiary center, 51% developed new morbidity based on FSS at hospital discharge. This cohort had high utilization of rehabilitation therapies and many required new technology at hospital discharge (129). In a larger cohort including survivors at 24 different PICUs, 24.8% of patients that required kidney replacement therapy had a new global functional disability at hospital discharge as determined by a change in POPC from baseline (OR 2.43) (42).

Our understanding of how AKI impacts functional outcomes and health-related quality of life in other at-risk populations, such as post cardiac surgery, remains incomplete. Pediatric survivors with low cardiac output post cardiac surgery have lower functional abilities and worse health-related quality of life at age 4 compared to those without low cardiac output in the post-operative period (130, 131). Although there is a known causal linkage between low cardiac output syndrome and AKI, there is a paucity of research on the independent association of AKI and long-term outcomes in survivors of congenital cardiac surgery. Rigorous ongoing research into long-term functioning and health-related quality of life for patients with congenital cardiac disease should include exposure to other risk factors, such a severity and number of episodes of cardiac surgery associated AKI.

### Longitudinal Impact on Adult Health and Life Course Outcomes

Young adults, aged 16–25 years, are a unique population whose physiology is not that of a child nor that of an aging adult. Unlike neonates, who are often at a higher risk of AKI due to their immature nephron function, and adults, who are at higher risk of AKI due to comorbidities, the young adult age group is typically thought to be healthy. However, it has been shown that even in critically ill patients aged 16–25 years admitted to a general adult ICU, the frequency of AKI (40%) exceeds that of the general PICU population (4, 132). Furthermore, in young adults the development of AKI in the ICU was found to be a significant predictor of hospital and ICU mortality, as well as mortality 1 year after discharge (132).

Young adults with congenital heart disease represent a particularly vulnerable group for AKI and its consequences, given their risk for repeated AKI events across a lifetime (133, 134). Importantly, the young adult congenital heart disease population is a growing population due to advances in cardiac care (135). In young adults with congenital heart disease that are diagnosed with AKI in the Cardiac ICU and have persistent kidney dysfunction 7–28 days after hospital discharge, there is a 12-fold increased odds of mortality at 5 years, independent of illness severity (134).

## Conclusions and Future Research Directions

Pediatric critical illness is frequently a dynamic state of complex interactions between every organ in the body responding and reacting to one another. Although once thought to be an isolated syndrome, it is clear that the implications of AKI have far reaching consequences that significantly affect ICU morbidity and mortality, as well as long term quality of life in survivors. Recent evidence has begun to elucidate how non-renal organs may be impacted by changes in fluid balance and the proinflammatory state secondary to the resulting AKI in critical illness. AKI is associated with both an immune dysregulated state and a proinflammatory state. The altered cytokine signature and endothelial dysfunction mediate most organ crosstalk in AKI, including brain and lung dysfunction. Abnormal cellular energy metabolism, similar to acute myocardial ischemia, can be demonstrated in the myocardium in AKI. Interestingly, the short and long term impact of AKI seems to have a sex predilection, with females being relatively protected from progression to CKD and dialysis.

In addition to the acute effects of AKI on other organ function, AKI impacts survivor functional outcomes, health-related quality of life, growth, and post-discharge mortality. Survivors of AKI represent a vulnerable population that require long-term, multi-disciplinary follow-up regardless of their discharge renal function. Prospective cohort studies designed to better understand the long-term impact of AKI on childhood development and growth are needed. Future research should focus on the identification of the mediators of organ cross-talk between the kidneys and the brain, heart, lung, and the immune system.

## Author Contributions

All authors listed have made a substantial, direct, and intellectual contribution to the work and approved it for publication.

## Conflict of Interest

The authors declare that the research was conducted in the absence of any commercial or financial relationships that could be construed as a potential conflict of interest.

## Publisher's Note

All claims expressed in this article are solely those of the authors and do not necessarily represent those of their affiliated organizations, or those of the publisher, the editors and the reviewers. Any product that may be evaluated in this article, or claim that may be made by its manufacturer, is not guaranteed or endorsed by the publisher.
